# Tenascins in Retinal and Optic Nerve Neurodegeneration

**DOI:** 10.3389/fnint.2017.00030

**Published:** 2017-10-23

**Authors:** Jacqueline Reinhard, Lars Roll, Andreas Faissner

**Affiliations:** Department of Cell Morphology and Molecular Neurobiology, Faculty of Biology and Biotechnology, Ruhr-University Bochum, Bochum, Germany

**Keywords:** extracellular matrix, glaucoma, glycoprotein, neurodegeneration, optic nerve, retina, tenascin-C, tenascin-R

## Abstract

Tenascins represent key constituents of the extracellular matrix (ECM) with major impact on central nervous system (CNS) development. In this regard, several studies indicate that they play a crucial role in axonal growth and guidance, synaptogenesis and boundary formation. These functions are not only important during development, but also for regeneration under several pathological conditions. Additionally, tenascin-C (Tnc) represents a key modulator of the immune system and inflammatory processes. In the present review article, we focus on the function of Tnc and tenascin-R (Tnr) in the diseased CNS, specifically after retinal and optic nerve damage and degeneration. We summarize the current view on both tenascins in diseases such as glaucoma, retinal ischemia, age-related macular degeneration (AMD) or diabetic retinopathy. In this context, we discuss their expression profile, possible functional relevance, remodeling of the interacting matrisome and tenascin receptors, especially under pathological conditions.

## Introduction

Numerous studies demonstrate that retina and optic nerve degeneration is highly associated with remodeling of various extracellular matrix (ECM) components. Glycoproteins and proteoglycans that surround retinal cells and optic nerve fibers represent major constituents of the ECM meshwork, known as the matrisome (Reinhard et al., 2015; Naba et al., 2016; Vecino et al., 2016). Various components of the matrisome came into focus as “good cop, bad cop” in de- and regeneration processes after injury or damage of the optic nerve (Isenmann et al., 2003; Ahmed et al., 2005; Ren et al., 2015). Additionally, remodeling of matricellular proteins is evident in the trabecular pathway, for instance in glaucoma pathogenesis (Wallace et al., 2015). In this review article, we focus on tenascin glycoproteins, which raised considerable attention in the context of degenerative processes in the retina and optic nerve.

### The Tenascin Family

In vertebrates, the family of tenascins comprises the four members tenascin-C, -R, -W and -X (Chiquet-Ehrismann and Tucker, 2011; Chiquet-Ehrismann et al., 2014). Expression of tenascin-R (Tnr) is restricted to the nervous system, whereas tenascin-C (Tnc) can also be found in non-nervous tissue. Due to the fact that little, if anything, has been reported about the role of tenascin-W and -X in the diseased visual system, this review article mainly focuses on the expression and functional importance of Tnc and Tnr in retinal and optic nerve degeneration and various eye diseases.

**Figure 1:**
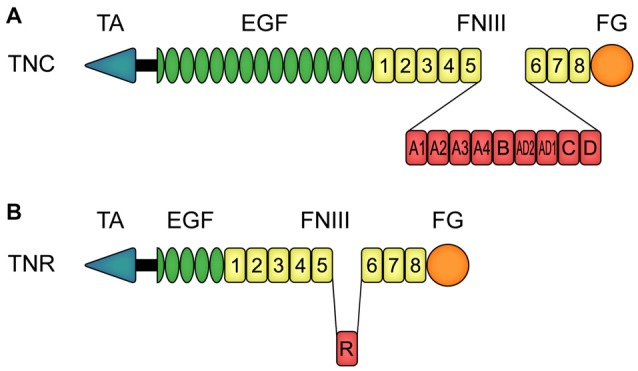
Modular assembly of human tenascin-C (TNC) and tenascin-R (TNR) monomers. **(A)** In human, each TNC monomer consists of an amino-terminal tenascin assembly (TA) domain, a cysteine-rich domain, 14.5 epidermal-growth factor (EGF)-like domains, eight constitutive fibronectin (FN)-type III homologous domains and a fibrinogen (FG)-like carboxy-terminal part. Between the FN-type III domains 5 and 6, TNC can carry the additional FN-type III domains A1, A2, A3, A4, B, AD2, AD1, C and D due to alternative splicing. **(B)** Human TNR also consists of a TA domain and a cysteine-rich domain, in this case followed by 4.5 EGF-like domains, eight constitutive FN-type III domains and a FG-like carboxy-terminal part. The alternatively spliced FN-type III domain R can be inserted between the FN-type III domains 5 and 6. Abbreviations: EGF, epidermal-growth factor-like domain; FG, fibrinogen-like domain; FNIII, fibronectin-type III homologous domain; TA, amino-terminal tenascin assembly domain; TNC, human tenascin-C; TNR, human tenascin-R.

Both tenascin molecules exhibit a modular structure (Nies et al., 1991; Siri et al., 1991; Jones F. S. and Jones, 2000; Jones P. L. and Jones, 2000; Joester and Faissner, 2001; Midwood and Orend, 2009). Tnc is an oligomeric protein, which consists of six monomers that are connected via a tenascin assembly (TA) domain at the amino-terminal region (Figure 1A). This constitution is also called hexabrachion. In human, each TNC monomer consists of a TA domain, followed by a cysteine-rich domain, 14.5 epidermal-growth factor (EGF)-like domains, eight fibronectin (FN)-type III domains and a fibrinogen (FG)-like carboxy-terminal part. Additional FN-type III domains, termed A1, A2, A3, A4, B, AD2, AD1, C and D, can be inserted between domain 5 and 6 (Spring et al., 1989; Nies et al., 1991; Dörries and Schachner, 1994; Joester and Faissner, 1999, 2001; Tucker et al., 2006). Via alternative *TNC* mRNA splicing, and based on a binary combinatorial potential, the generation of up to 512 isoforms in humans is feasible (Joester and Faissner, 1999, 2001; Theocharidis et al., 2014; Midwood et al., 2016; Faissner et al., 2017). One interesting feature of Tnc is that it exhibits both adhesive and anti-adhesive properties (Faissner and Kruse, 1990; Chiquet-Ehrismann et al., 1991; Faissner, 1997). For instance, the FN-type III region exhibits pro-adhesive characteristics, whereas the EGF-like domains show anti-adhesive properties (Spring et al., 1989; Ajemian et al., 1994; Gotz et al., 1996). The latter are also associated with proliferation, growth cone repulsion and migration (Joester and Faissner, 2001; Swindle et al., 2001; Loers and Schachner, 2007). Human TNR exhibits a similar modular structure, but it is composed of three monomers (Figure 1B; Schachner et al., 1994). Each monomer consists of an amino-terminal TA domain, a cysteine-rich domain, 4.5 EGF-like repeats, eight or nine FN-type III domains and a FG carboxy-terminal part.

### Tenascins in the Developing and Adult Healthy Retina and Optic Nerve

**Figure 2:**
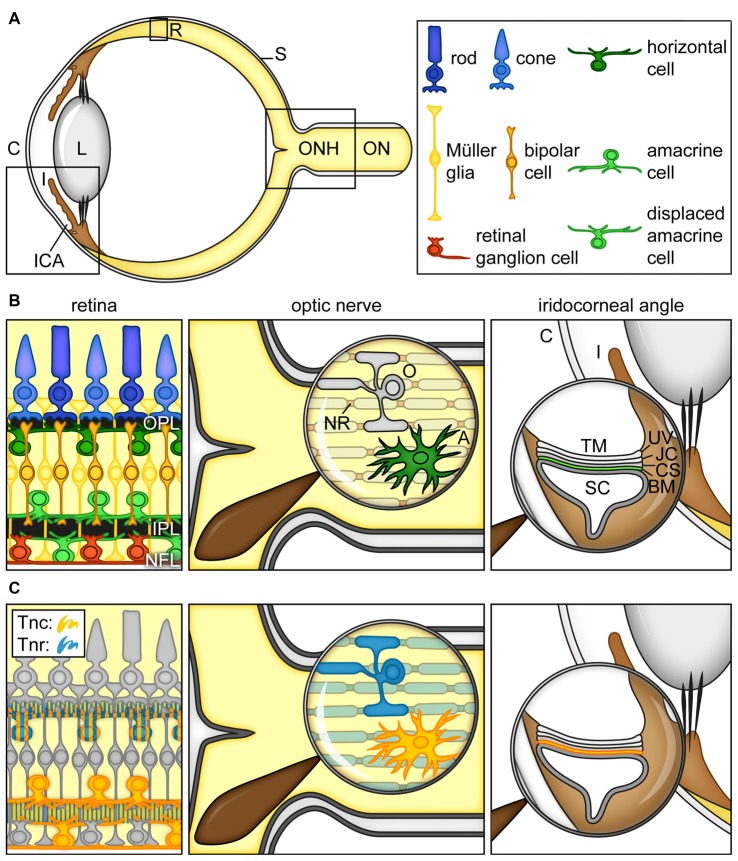
Cartoon summarizing the current view on the expression of tenascins in the retina, optic nerve and iridocorneal angle. **(A)** Scheme of the adult human eye. **(B)** Scheme of the retina, optic nerve and iridocorneal angle.** (C)** Visualization of Tnc- and Tnr-expressing cell types/structures in the retina, optic nerve and iridocorneal angel. In the retina, horizontal, amacrine and displaced amacrine cells are a main source of Tnc expression (orange). Horizontal cells also co-express large amounts of Tnr (blue). Additionally, signals of both proteins can be observed in the plexiform layers (orange/blue). In the optic nerve, astrocytes show a strong Tnc expression (orange). Tnr (blue) is highly expressed by optic nerve oligodendrocytes and localized at myelinated fibers and nodes of Ranvier. In the iridocorneal angle, the basement membrane underlying the inner wall of Schlemm’s canal contains Tnc protein (orange). Abbreviations: A, astrocyte; BM, basement membrane; C, cornea; CS, corneoscleral tissue; I, iris; ICA, iridocorneal angle; IPL, inner plexiform layer; JC, juxtacanalicular tissue; L, lens; NFL, nerve fiber layer; NR, node of Ranvier; O, oligodendrocyte; ON, optic nerve; ONH, optic nerve head; OPL, outer plexiform layer; R, retina; S, sclera; SC, Schlemm’s canal; TM, trabecular meshwork; Tnc, tenascin-C; Tnr, tenascin-R, UV, uveal tissue.

As part of the eye (Figure 2A), the retina and the optic nerve develop from neuroectodermal tissue. During retinogenesis, seven main cell types, namely retinal ganglion cells (RGCs), amacrine, bipolar, horizontal, Müller glia as well as cone and rod photoreceptor cells, arise from multipotent retinal progenitor cells in highly conserved and overlapping waves (Cepko et al., 1996; Dyer and Cepko, 2001; Marquardt, 2003; Agathocleous and Harris, 2009; Heavner and Pevny, 2012). Until adulthood, following a maturation and synaptic fine-tuning period, retinal cell nuclei are assigned to specific nuclear layers, while their synaptic processes are arranged in plexiform and nerve fiber layers (Figure 2B).

Indeed, the retina is an excellent model system to study developmental aspects such as proliferation and differentiation, but also axonal growth and guidance as well as pathfinding (McLaughlin et al., 2003; Oster et al., 2004). Nasal and temporal projections from the retina are transmitted via RGC axons, which form the optic nerve. RGC axons from both eyes converge in the optic chiasm at the base of the hypothalamus and segregate into ipsi- and contralaterally projecting fibers (Petros et al., 2008; Erskine and Herrera, 2014). Via the optic tract, axons project in a highly topographic manner into subcortical and cortical areas to transfer the visual information.

Intrinsic and extrinsic factors, which comprises transcription factors, growth factors and a variety of ECM components, including tenascins, influence retinogenesis and the growth of optic nerve fibers (Thanos and Mey, 2001; Hatakeyama and Kageyama, 2004; Harada et al., 2007; Agathocleous and Harris, 2009; Xiang, 2013; Reinhard et al., 2015).

In the developing retina, Tnc becomes detectable within the inner neuroblastic layer at embryonic day 13 (Klausmeyer et al., 2007). In the adult retina, it is synthesized by different neuronal subtypes, including horizontal, amacrine and displaced amacrine cells and is prominently enriched in the outer and inner plexiform as well as in the nerve fiber layer (D’Alessandri et al., 1995; Sánchez-López et al., 2004; Figure 2C). As shown by Siddiqui et al. (2009), cultivated postnatal Müller glia cells also express large Tnc isoforms. In the optic nerve, astrocytes secrete huge amounts of the Tnc protein (Bartsch et al., 1995; D’Alessandri et al., 1995; Garwood et al., 2004; Reinhard et al., 2015).

Tnr, also known as janusin/J1-160/180 in rodents or restrictin in the chicken, is initially expressed upon postnatal stages in the developing retina and optic nerve (Ffrench-Constant et al., 1988; Bartsch et al., 1993; Wintergerst et al., 1993; Joester and Faissner, 2001). Later, Tnr expression peaks until the third postnatal week and then decreases again. In the adult retina, horizontal cells are the main cellular source of Tnr (Figure 2C). Due to the proximity of Tnr-expressing cells, large amounts of protein are found in the outer plexiform layer. Nevertheless, the inner plexiform and nerve fiber layer also show detectable levels of the Tnr protein, suggesting low expression by other retinal cell types or intraretinal protein transport. In the optic nerve, it is highly expressed by oligodendrocytes and associated with myelinated fibers and nodes of Ranvier with ongoing age until adulthood. In contrast, Tnr is absent from the unmyelinated proximal, retina-near part of the optic nerve.

## Role of Tenascins in Eye Diseases

In the central nervous system (CNS), Tnc exhibits high expression during early development. With ongoing maturation, it is progressively downregulated, but re-expressed under pathological conditions (Garwood et al., 2004; Roll et al., 2012; Reinhard et al., 2015). The role of Tnc remodeling in the neural stem/progenitor compartment has been reviewed comprehensively (Roll and Faissner, 2014; Theocharidis et al., 2014; Faissner and Reinhard, 2015; Faissner et al., 2017). It regulates proliferation and differentiation and is also enriched in the adult neural stem cell niche. Additionally, Tnc is involved in barrier formation, for example in the barrel cortex during development and as a constituent of the glial scar after injury. Also in many cancers, Tnc is highly expressed and promotes migration as well as angiogenesis (Orend and Chiquet-Ehrismann, 2006; Midwood and Orend, 2009; Brösicke and Faissner, 2015; Reinhard et al., 2016). These examples show the huge spectrum of—in part ambivalent—Tnc-mediated functions. Furthermore, various studies suggest that tenascin glycoproteins might be involved in degenerative processes of the retina and optic nerve as well as eye diseases e.g., glaucoma.

### Tenascin-C in Glaucoma

Glaucoma is one of the leading causes of visual impairment and irreversible blindness worldwide. It is a neurodegenerative disease characterized by morphological changes of the optic nerve head and retinal nerve fiber layer as well as progressive RGCs loss (EGS, 2017). In 2010, approximately 4.2 million people were visually impaired due to glaucoma (Bourne et al., 2016). This number will likely rise to about 11.2 million people by 2020 (Quigley and Broman, 2006). Among others, age, genetic predisposition and intraocular pressure (IOP) elevation are considered the most important risk factors for glaucoma. However, its pathophysiology is still poorly understood.

Various studies indicate that remodeling of Tnc is strongly associated with high-pressure glaucoma (Table 1). Pena et al. (1999) recognized that enhanced Tnc expression is associated with reactive astrocytes in the human optic nerve head of primary open-angle glaucoma (POAG) patients. Although the precise function of Tnc in glaucoma disease is still unknown, it was assumed that it might act as barrier molecule, which locally restricts detrimental humoral and blood-derived factors, to protect RGC axons. Along these lines, Johnson et al. (2007) described a prominent Tnc upregulation in the pressure-injured optic nerve head of a rat ocular hypertension glaucoma model. Additionally, Tnc might be involved in reactivation of astrocytes, which play a crucial role in glaucomatous optic nerve fibrosis (Schneider and Fuchshofer, 2016).

**Table 1:** Summary of the current knowledge on the regulation of tenascin-C (Tnc) and tenascin-R (Tnr) expression in retinal and optic nerve degeneration and eye diseases.

	Type of degeneration/eye disease	References
**Tnc**	**AMD**	
	High levels in choroidal neovascular membranes of AMD patients	Nicolò et al. (2000), Fasler-Kan et al. (2005), Afshari et al. (2010) and Kobayashi et al. (2016b)
	**Diabetic retinopathy**	
	Upregulated in fibrovascular membranes in eyes of diabetic patients	Ishikawa et al. (2015) and Kobayashi et al. (2016a)
	Upregulated in basement membranes of diabetic human eyes	To et al. (2013)
	Upregulation in intravitreal membranes of patients with proliferative traumatic, idiopathic vitreoretinopathy and proliferative diabetic retinopathy	Hagedorn et al. (1993)
	**Glaucoma**	
	Upregulated in the retina and optic nerve of an autoimmune-glaucoma rat model	Reinehr et al. (2016b)
	Upregulated in the ONH of an IOP-induced glaucoma rat model	Johnson et al. (2007)
	Upregulation of specific isoforms in mechanically stretched TM cells	Keller et al. (2007)
	Upregulated in the ONH of POAG patients	Pena et al. (1999)
	**Retinal ischemia**	
	Downregulation of small isoforms in the retina of an ischemia/reperfusion rat model	Reinhard et al. (2017)
	**Optic nerve de-/regeneration**	
	Upregulated following optic nerve crush in the goldfish	Battisti et al. (1995)
	Upregulated after rat optic nerve transection	Ajemian et al. (1994)
**Tnr**	**Retinal ischemia**	
	Upregulation of the large isoform in the retina in a rat ischemia/reperfusion model	Reinhard et al. (2017)
	**Optic nerve de-/regeneration**	
	Upregulated in the regenerating visual pathway of the lizard	Lang et al. (2008)
	Expression not altered in the optic nerve of mice following injury	Becker et al. (2000)
	Reduced expression levels in the optic nerve of the salamander	Becker et al. (1999)

*Abbreviations: AMD, age-related macular degeneration; ONH, optic nerve head; POAG, primary open-angle glaucoma; TM, trabecular meshwork; Tnc, tenascin-C; Tnr, tenascin-R*.

IOP rises due to impaired aqueous humor outflow via the trabecular pathway in the iridocorneal angle (Abu-Hassan et al., 2014; Dautriche et al., 2014). In this regard, it is interesting to note that Tnc is an extracellular component within the human juxta-canalicular tissue (JCT). In addition, Tnc was detected in trabecular meshwork (TM) cells (Ueda and Yue, 2003; Pattabiraman and Rao, 2010; Keller et al., 2013; Figure 2B). In the JCT, Tnc is predominantly localized in basement membranes underlying the inner wall of Schlemm’s canal (Figure 2C).

An abnormal accumulation of ECM constituents increases aqueous humor outflow resistance through the trabecular pathway (Gabelt and Kaufman, 2005). The functional importance of matricellular protein production and turnover to control outflow resistance in the TM has been reviewed (Wallace et al., 2014, 2015; Tamm et al., 2015). Several matricellular proteins, including the connective tissue growth factor, thrombospondin, Tnc and Tnx, appear to play a role in TM fibrosis. For instance, deficiency of the matrix glycoproteins thrombospondin 1 and SPARC (secreted protein acidic and rich in cysteine) has been shown to enhance outflow facility and lower IOP in mouse models of glaucoma (Wallace et al., 2015).

Keller et al. (2007) noted increased levels of Tnc in response to mechanical stretching of porcine TM cells in a perfusion culture model. Isoforms of Tnc identified in those TM cells included FN-type III 5-D-6, 5-6, A1-B as well as B-D-6. Interestingly, levels of Tnc FN-type III domain D transcripts were also elevated due to mechanical stretching of TM cells, indicating changes in alternative splicing that might affect TM cell-ECM interaction.

The effects of Tnc knockdown on TM outflow resistance were studied in more detail in anterior segment perfusion organ cultures (Keller et al., 2013). Here, Tnc was upregulated in response to IOP elevation. Nevertheless, the outflow rate was not altered by Tnc-silencing in anterior segments following IOP elevation. In addition, IOP was not altered in Tnc knock-out compared to control mice, indicating that Tnc does not directly contribute to the regulation of outflow resistance. However, Yang et al. (2016) described the effects of induction and inhibition of matrix cross-linking on remodeling of the aqueous humor outflow resistance by TM cells. In this study, genipin, a potent inducer of ECM crosslinking and inhibitor of aqueous humor outflow, reduced the levels of Tnc and other ECM components such as collagen I, elastin and the chondroitin sulfate proteoglycan (CSPG) versican. These findings indicate that changes in the ECM composition, crosslinking and turnover are highly dynamic and influence outflow resistance. Recently, the group around Kuehn showed a positive effect on outflow facility after transplantation of induced pluripotent stem cell-derived TM cells into a glaucoma model. Since the transplanted cells do not persist for long, it was theorized that the cells might alter TM conditions (Zhu et al., 2016, 2017). These alterations could also be related to changes in the ECM. The TM serves as an important target for the treatment of IOP elevation in glaucoma (Ferrer, 2006). In a future perspective, further knowledge of ECM-TM physiology is necessary to develop novel and powerful IOP lowering therapies.

Tenascins are also key regulators of the immune system and neuroinflammatory processes (Jakovcevski et al., 2013). A complex interplay and functional relationship between neural and immune cells in various autoimmune diseases, e.g., multiple sclerosis and neuropathies, are evident. Tnc was reported as one major ECM component, which modulates transforming growth factor β (TGFβ)/Smad signaling and myofibroblast generation during wound healing of the corneal stroma (Saika et al., 2016). Also in the TM, TGFβ increases outflow resistance via alteration of ECM homeostasis and cell contractility (Fuchshofer and Tamm, 2012; Prendes et al., 2013; Wang et al., 2017). In addition, Tnc was previously reported to influence the immune system through the toll-like receptor 4 (TLR4; Midwood et al., 2009). It promotes an inflammatory response via macrophage generation, activation of TLR4 and the secretion of proinflammatory cytokines after stimulation with lipopolysaccharide (Piccinini and Midwood, 2012; Piccinini et al., 2016). Indeed, Tnc deficiency protects mice from experimental autoimmune encephalomyelitis and plays a key role in pathogenesis of CNS autoimmunity (Momcilovic et al., 2017). Interestingly, various studies have shown that *TLR4* gene polymorphisms are associated with an increased risk of glaucoma (Shibuya et al., 2008; Navarro-Partida et al., 2017a,b). This confirms that TLR4-mediated signaling is involved in this disease.

Several studies have also investigated the possible involvement of the immune system in glaucoma pathogenesis (Tezel and Wax, 2004; Tezel, 2009; Rieck, 2013; Ramirez et al., 2017). Recently, we have noticed Tnc dysregulation in an IOP-independent, experimental autoimmune glaucoma model (Reinehr et al., 2016b). In this glaucoma model, RGC loss, optic nerve damage, reactive gliosis as well as complement activation have been described (Joachim et al., 2013, 2014; Casola et al., 2015; Noristani et al., 2016; Reinehr et al., 2016a). Furthermore, upregulation of Tnc and the CSPG phosphacan, an interaction partner of Tnc, was found in the retina and optic nerve of the autoimmune glaucoma model (Reinehr et al., 2016b). Most interestingly, elevated Tnc levels were observed before RGC loss occurred in this model. Regarding this finding, Tnc might act as an early indicator of glaucomatous neurodegeneration, although the function of Tnc in IOP-independent glaucoma is not well understood yet.

### Tenascin-C and Tenascin-R in Retinal Ischemia

Ischemia represents a common pathomechanism in several retinal diseases, like age-related macular degeneration (AMD), diabetic retinopathy, glaucoma and retinal vascular occlusion (Mizener et al., 1997; Coleman et al., 2013; Sim et al., 2013).

Several studies reported on a dysregulation of Tnc following cerebral, hepatic as well as myocardial ischemia (Lu et al., 2003; Taki et al., 2010, 2015; Kuriyama et al., 2011). We recently analyzed the regulation of ECM glycoproteins and proteoglycans in the retina and optic nerve of an ischemia/reperfusion rat model (Reinhard et al., 2017; Table 1). An interesting finding of this study includes the prominent upregulation of several CSPGs in the ischemic optic nerves. Furthermore, in the retina, elevated levels of the large Tnr isoform were found, while reduced levels of smaller Tnc isoforms were observed after ischemia/reperfusion. These findings support the idea of an isoform-dependent regulation of tenascins. In future studies, domain-specific Tnc antibodies (Brösicke et al., 2013; Reinhard et al., 2016) should be used to relate specific isoforms to distinct retinal cell types under pathological conditions.

In the CNS, tenascins represent main structural and functional constituents of synaptic sites (Dityatev et al., 2010; Kwok et al., 2011; Heikkinen et al., 2014; Dzyubenko et al., 2016; Song and Dityatev, 2017). Also in the retina, tenascins are highly associated with synaptic layers (Bartsch et al., 1993; D’Alessandri et al., 1995; Wahlin et al., 2008). We have previously shown a co-localization of Tnc and synaptophysin in the healthy retina (Reinhard et al., 2015). In sum, the dysregulation of tenascins after retinal ischemia might reflect the response or damage of retinal neurons or synaptic reorganization.

### Tenascin-X and Tenascin-C in Age-Related Macular Degeneration

AMD is defined by a deterioration of the macula and represents a major cause of vision impairment worldwide (Jager et al., 2008; Ding et al., 2009; Lim et al., 2012). It is a multifactorial disease that affects primarily photoreceptor cells, retinal pigment epithelium (RPE), Bruch’s membrane as well as choriocapillaries (Bhutto and Lutty, 2012). Additionally, AMD is characterized by extracellular depositions between Bruch’s membrane and the RPE, termed drusen, which includes complement components, glycoproteins and lipids (Crabb, 2014; Fernandez-Godino et al., 2016). Choroidal neovascularization is the defining characteristic of wet AMD.

The tenascin family member Tnx was identified in AMD patients in a genome-wide association study (Cipriani et al., 2012). In a plasma protein screen to identify biomarker, Tnx was differentially expressed in AMD patients compared to the healthy controls (Kim et al., 2014).

Additionally, high levels of Tnc were observed in choroidal neovascular membranes from AMD patients (Nicolò et al., 2000; Fasler-Kan et al., 2005; Afshari et al., 2010; Kobayashi et al., 2016b; Table 1). Here, RPE cells restricted to scar areas exhibited a strong staining for Tnc. Tnc was also identified as a candidate to cause RPE adhesion failure in damaged and aged Bruch’s membrane. In this regard, Afshari et al. (2010) described that Tnc inhibits RPE attachment and migration. Interestingly, this inhibition can be overcome via integrin activation or expression of Tnc-binding integrin α9, which allows RPE cells to interact with the AMD-affected Bruch’s membrane (Afshari et al., 2010). Additionally, Tnc secretion by transdifferentiated RPE cells is considered to promote choroidal neovascular membrane formation via integrin αv in a paracrine manner (Kobayashi et al., 2016b). Here, Tnc was discussed as potential target for the inhibition of choroidal neovascular membrane formation in AMD.

### Tenascin-C in Diabetic Retinopathy

Diabetic retinopathy is also highly associated with retinal vascular dysfunction. Tnc was found in intravitreal membranes of patients with traumatic and idiopathic proliferative vitreoretinopathy as well as in diabetic retinopathy (Hagedorn et al., 1993; Table 1). In light of these results, it was suggested that Tnc likely controls cellular adhesion and ECM formation under pathological conditions. Structural, morphological as well as biophysical changes of ocular vasculature basement membranes were reported to be accompanied by ECM remodeling (To et al., 2013). Here, a higher Tnc expression was detected in basement membranes of diabetic compared to non-diabetic human eyes. Additionally, Tnc was reported to be involved in inflammatory processes of diabetic retinopathy. Increased Tnc levels were found in retinal endothelia cells following tumor necrosis factor α and interleukin 1β stimulation (Palenski et al., 2013). Expression analysis in fibrovascular membranes from patients with proliferative diabetic retinopathy revealed an upregulation of Tnc (Ishikawa et al., 2015). Recently, Kobayashi et al. (2016a) showed that Tnc, secreted from vascular smooth muscle cells, promotes angiogenesis in fibrovascular membranes associated with diabetic retinopathy.

## Role of Tenascin-C and Tenascin-R in Optic Nerve Injury, Degeneration and Regeneration

RGC nerve fibers exhibit a poor regeneration capacity after injury, which often leads to irreversible vision loss. Therefore, multiple studies focused on the improvement of RGC survival as well as axonal regrowth, guidance and pathfinding (Fischer and Leibinger, 2012; Crair and Mason, 2016). Indeed, the optic nerve serves as an ideal research model to follow axonal de- and regeneration processes and RGC survival in order to develop novel therapeutic strategies, for instance after glaucomatous damage (Diekmann and Fischer, 2013; Gauthier and Liu, 2016; Calkins et al., 2017; Tamm and Ethier, 2017). Over the past decades, it has become evident that regeneration capacity differs a lot with age and between various species. Regeneration is more efficient in lower compared to higher vertebrates.

After optic nerve damage, Wallerian degeneration, demyelination, immune activation and glial scar formation can be observed. In this context, it has become evident that next to the intrinsic cellular repertoire, an inhibitory environment prevents regrowth of optic nerve fibers (Fischer, 2012). ECM proteins are main components of this inhibitory environment. Here, tenascins were described as crucial boundary formation molecules in optic nerve degeneration. Those boundaries represent important decision breakpoints to navigate growing axons during development as well as following injury or disease (Silver, 1994). In the adult mammalian CNS, after injury, Tnc and Tnr play opposing roles in regeneration of optic nerve fibers, with Tnc being promotive and chemo-attractive, while Tnr plays an inhibitory and chemo-repulsive role (Jakovcevski et al., 2013). The current knowledge on the regulation of Tnc and Tnr following optic nerve degeneration and regeneration is also summarized in Table 1.

Compared to mammals, the CNS of the zebrafish displays a robust axonal regeneration capacity and allows visualization of axonal regeneration and re-myelination *in vivo*. Tnr was also described as a repulsive guidance molecule of newly growing as well as regenerating optic nerve fibers in the zebrafish (Becker and Becker, 2002; Becker et al., 2004). Becker et al. (2000) reported that Tnr inhibits regrowth of optic nerve fibers *in vitro*. In contrast to the reduced Tnr expression levels observed in the optic nerve of the salamander (Becker et al., 1999), it persists in the optic nerve of mice following injury (Becker et al., 2000). Due to the continued expression, it was suggested that Tnr inhibits axonal regeneration *in vivo*. In addition, Tnr and axon growth-promoting molecules were found upregulated in the regenerating visual pathway of the lizard *Gallotia galloti* (Lang et al., 2008).

Since Tnr is highly associated with myelinated optic nerve fibers and nodes of Ranvier, it was proposed that it might have a functional relevance in myelination processes. Recordings of action potentials from Tnr knock-out mice revealed reduced axonal conduction velocities compared to control mice. In contrast, no significant differences in the number of myelinated optic nerve fibers or in the myelin ultrastructure were observed in Tnr knock-out compared to wild-type mice (Weber et al., 1999).

A potential role of Tnc in neural repair of the injured rat optic nerve was initially reported by Ajemian et al. (1994). Here, after optic nerve transection, Tnc immunoreactivity appeared in astrocytes at the border of the injury. Additionally, it was proposed to act as important barrier molecule for oligodendrocyte precursor migration during development (Bartsch et al., 1994; Kiernan et al., 1999). Following crush injury of the goldfish optic nerve, Tnc was reported to be associated with activated granular macrophages, although its expression in activated astrocytes and microglia was also assumed (Battisti et al., 1995). In contrast, although Bernhardt et al. (1996) described lesion-induced upregulation of several glia cell-associated genes after axotomy in the adult zebrafish, Tnc levels were not altered. In the embryonic and postnatal rat retina Tnc promotes axonal outgrowth, especially via the alternatively spliced FN-type III D domain (Siddiqui et al., 2008).

## Tenascin Signaling and the Interacting Matrisome Under Pathological Conditions

A huge diversity of interacting molecules can be observed for the tenascin proteins. For Tnc this includes the cell adhesion molecules contactin-1 (Rigato et al., 2002; Czopka et al., 2010), various CSPGs of the lectican family such as aggrecan and neurocan, phosphacan/receptor protein tyrosine phosphatase β/ζ (RPTPβ/ζ; Barnea et al., 1994; Milev et al., 1997; Rauch et al., 1997; Garwood et al., 1999; Adamsky et al., 2001; Lundell et al., 2004) but also several integrin family members like α2β1, α7β1, α8β1, α9β1 and αvβ3 (Tucker and Chiquet-Ehrismann, 2015; Faissner et al., 2017).

The signaling of integrins in RGC-glia interactions is crucial for RGC survival and process extension (Vecino et al., 2016). In the developing retina, β1 integrins mediate RGC neurite outgrowth and α integrin-subunits are expressed in RGCs. Tnc inhibits axonal growth, but also displays axon growth-promoting properties, when appropriate receptors like α9β1 integrin are expressed. The FN-type III domain Nr. 3 of Tnc is a ligand of α9β1 integrin (Yokosaki et al., 1998). However, in the adult, neurite outgrowth-promoting α9β1 integrin is absent in neurons, which counteracts regeneration properties (Wang et al., 1995). Interestingly, in the spinal cord, regeneration of sensory axons can be achieved by expression of Tnc-binding α9 integrin and kindlin-1 (Andrews et al., 2009; Cheah et al., 2016). As recently reported, co-transduction of α9 integrin and the integrin activator kindlin-1 represents a promising approach to promote optic nerve regeneration (Fawcett, 2017). Integrin-Tnc signaling might also play a role in the glaucomatous optic nerve head, as Morrison reported on the crucial importance of integrins in optic neuropathy (Morrison, 2006).

As mentioned above, CSPGs are major interaction partners of Tnc. In the CNS, CSPGs are widely recognized as major inhibitory constituents of the glial scar (Silver and Silver, 2014). Notably, elevated levels of the CSPGs aggrecan, brevican and phosphacan were noted in the optic nerve after retinal ischemia (Reinhard et al., 2017). Following laser lesion, differential expression of RPTPβ/ζ was observed in the retina (Besser et al., 2009). In the non-injured retina of Tnc deficient mice, an upregulation of the DSD-1 epitope, recognized by the monoclonal antibody 473HD and localized on phosphacan/RPTPβ/ζ, was revealed when compared to the wild-type mice (Besser et al., 2012). Since RPTPβ/ζ knock-out mice show a disturbance of Müller glia processes, RPTPβ/ζ might be implicated in the assembly of the retinal structure (Horvat-Bröcker et al., 2008). There is also strong evidence that Tnc interacts with a variety of growth factors. In this context, it has been shown that the FN-type III domain 5 of Tnc displays a high binding affinity for the fibroblast growth factor 2 (FGF2), neurotrophin-3, platelet-derived growth factor-BB as well as TGFβ1 (De Laporte et al., 2013). After brain lesion, TGFβ and FGF2 control Tnc expression in astrocytes and reactive cells (Smith and Hale, 1997; Dobbertin et al., 2010). Therefore, Tnc might contribute to the signaling environment after lesion. Nevertheless, the direct functional contribution of Tnc has not been elucidated yet. Tnc was found to enhance FGF2 sensitivity of de-differentiating Müller glia cells *in vitro*. Furthermore, Tnc knock-out mice show an impaired de-differentiation capacity (Besser et al., 2012).

Likewise, Tnc displays a complex interactome with other ECM glycoproteins. For instance, its interaction with fibronectin and Tnr was reported (Chiquet-Ehrismann et al., 1991; Chung et al., 1995; Probstmeier et al., 2000; Giuffrida et al., 2004). After CNS damage, glial-released fibronectin exhibits neuroprotective repair function and promotes outgrowth of neurites *in vitro* (Tom et al., 2004; Tate et al., 2007; Kim et al., 2013). Also the neural transmembrane protein CALEB (chicken acidic leucine-rich EGF-like domain-containing brain protein/neuroglycan C) directly interacts with Tnc and Tnr (Schumacher et al., 2001; Schumacher and Stübe, 2003). Interestingly, CALEB is highly expressed following optic nerve lesion (Schumacher et al., 2001; Schumacher and Stübe, 2003). CALEB expression is strongly associated with developing as well as regenerating RGCs.

The adhesion molecule contactin-1 was identified as an important neuronal receptor for Tnr. Interaction of these two molecules was reported to mediate the repulsion and defasciculation of neurites (Pesheva et al., 1993; Milev et al., 1998; Xiao et al., 1998). Additionally, as shown for Tnc, Tnr displays an overlapping expression pattern with the CSPG phosphacan (Xiao et al., 1997; Milev et al., 1998). Regarding these findings, Tnc and Tnr seem to represent key components of the retina and optic nerve matrisome under pathological conditions. Both molecules partially display an overlapping extracellular distribution and directly contribute to de- and regeneration processes.

## Conclusion

In the CNS, tenascin glycoproteins are important constituents of a highly regulated and dynamic matrisome. In sum, the current literature supports the notion that Tnc and Tnr are implicated in various pathological processes following retinal and optic nerve degeneration as well as various eye diseases (Table 1). Under pathological conditions, during development as well as regeneration, the opposed character of Tnc and Tnr is crucial for the growth and guidance of axons. In particular, the manipulation of Tnc-integrin signaling might be a promising approach to enhance axonal regeneration. Additionally, as a structural ECM component of the TM, Tnc and the interacting matrisome might be a target to improve IOP lowering therapies. We provide evidence that TLR4 signaling is involved in glaucoma development. Tnc upregulation observed under these conditions might indicate an immunomodulatory role, mediated by TLR4. Since tenascins are highly enriched at synaptic sites in the retina, it is plausible to speculate that they might play a role in synaptic remodeling, also under pathological conditions. Nevertheless, to verify these potential functions, further analyses have to be performed.

## Author Contributions

JR wrote the manuscript. LR designed the figures. LR and AF revised the manuscript. All authors have approved the final article.

## Conflict of Interest Statement

The authors declare that the research was conducted in the absence of any commercial or financial relationships that could be construed as a potential conflict of interest.

## Abbreviations

AMD: age-related macular degeneration
CNS: central nervous system
CSPG: chondroitin sulfate proteoglycan
ECM: extracellular matrix
FGF2: fibroblast growth factor 2
IOP: intraocular pressure
JCT: juxta-canalicular tissue
POAG: primary open-angle glaucoma
RGC: retinal ganglion cell
RPE: retinal pigment epithelium
RPTPβ/ζ: receptor protein tyrosine phosphatase β/ζ
TGF: transforming growth factor
TLR4: toll-like receptor 4
TM: trabecular meshwork
Tnc: tenascin-C
Tnr: tenascin-R.
